# Overexpression of KPC contributes to ceftazidime-avibactam heteroresistance in clinical isolates of carbapenem-resistant *Klebsiella pneumoniae*

**DOI:** 10.3389/fcimb.2024.1450530

**Published:** 2024-12-06

**Authors:** Yitan Li, Xiandi Chen, Yingyi Guo, Yingzhuo Lin, Xiaohu Wang, Guohua He, Mingzhen Wang, Jianbo Xu, Mingdong Song, Xixi Tan, Chao Zhuo, Zhiwei Lin

**Affiliations:** ^1^ Key Laboratory of Respiratory Disease, People’s Hospital of Yangjiang, Yangjiang, China; ^2^ Guangzhou Institute of Respiratory Health, First Affiliated Hospital of Guangzhou Medical University, Guangzhou, China

**Keywords:** ceftazidime-avibactam, carbapenem-resistant *Klebsiella pneumoniae*, heteroresistance, expression of KPC, inhibition strategy

## Abstract

Ceftazidime–avibactam (CZA) is one of the effective antibiotics used for the treatment of carbapenem-resistant *Klebsiella pneumoniae* (CRKP) infections, but its resistance rate has increased recently. Previous studies have focused on the mechanisms of CZA resistance, while its heteroresistance in CRKP remains poorly understood. This study aimed to investigate the characteristics and mechanisms of CZA heteroresistance in CRKP isolates. A total of 311 CRKP clinical strains were collected in China from 2020 to 2022. The MICs of CZA and other antibiotics against *K. pneumoniae* were determined by broth microdilution method. The occurrence of CZA heteroresistance in CRKP was evaluated with population analysis profiling (PAP) and their characteristics were detected by polymerase chain reaction (PCR). The underlying mechanism of CZA heteroresistance in CRKP strains was investigated by molecular sequencing, whole genome sequencing (WGS), quantitative real-time PCR (qRT-PCR), and *in vitro* functional experiments. Strategies for preventing the emergence of CZA heteroresistance and alternative treatment options for strains exhibiting CZA heteroresistance were further explored. Thirty-four (12.4%) CZA-susceptible CRKP isolates were found to exhibit heteroresistance to CZA. All heteroresistant strains belonged to KPC-2 (97.1%) or KPC-3 (2.9%). The dominant multilocus sequence typing (MLST) was ST11 (64.7%) and the prevalent capsular serotypes were KL47 (38.2%) and KL64 (32.4%). Imipenem-relebactam and meropenem-vaborbactam still exhibited excellent antimicrobial activity against the resistant subpopulations of CZA heteroresistant strains. No significant mutations were found in KPC, OmpK35/36, PBP2/3, and LamB in resistant subpopulations. The relative expression and copy number of *bla*_KPC_ were significantly upregulated in 47.1% and 35.3% of the resistant subpopulations compared with their parental strains, respectively. Silencing *bla*_KPC_ expression significantly decreased the CZA MIC in resistant subpopulations with high *bla*_KPC_ expression and hindered the emergence of CZA heteroresistance in their parental strains. Moreover, increasing the avibactam concentration to 8 or 16 mg/L or combining CZA with 0.5 × MIC tigecycline significantly suppressed the formation of CZA heteroresistance (*P*<0.05). In conclusion, we identified the occurrence of CZA heteroresistance in CRKP in China, which was attributed to the overexpression of KPC. Increasing the concentration of avibactam or combining CZA with tigecycline could effectively prevent the development of CZA heteroresistance in CRKP isolates. Besides, imipenem-relebactam and meropenem-vaborbactam may serve as alternative therapeutic options when clinical isolates with CZA heteroresistance are detected.

## 1 Introduction

*Klebsiella pneumoniae* is one of the most common gram-negative bacterial species responsible for severe infections such as pneumonia, urinary tract infection, septicemia, and meningitis (Hu et al., 2020). Carbapenems and other β-lactam antibiotics are commonly used antimicrobial agents in the treatment of *K. pneumoniae* infections. Due to the widespread use of carbapenem antibiotics, the prevalence of carbapenem-resistant *K. pneumoniae* (CRKP) strains has markedly increased worldwide in recent decades (Qin et al., 2020). The CRKP infections pose a serious threat to public health, and the therapeutic options available to treat these infections are limited because CRKP is resistant to almost all commonly used antibiotics (Lee et al., 2016). CZA is one of the most effective antibiotics for CRKP infections, making it an important tool to combat these infections.

Ceftazidime–avibactam (CZA) is a combination therapy consisting of the broad-spectrum cephalosporin, ceftazidime, and the β-lactamase inhibitor, avibactam (Sader et al., 2018). The latter is a novel class of reversible, covalent, non-β-lactam β-lactamase inhibitors that demonstrates broader spectrum enzyme inhibition, including Amber class A, C, and some class D β-lactamases, which are mainly responsible for the hydrolysis of β-lactam antibiotics (Giri et al., 2019). CZA exhibits excellent activity against carbapenem-resistant *Enterobacteriaceae* and *Pseudomonas aeruginosa*, especially demonstrates potent activity against KPC-producing *K. pneumoniae* strains, which are the most prevalent CRKP isolates in China (Barber et al., 2018). In February 2015, CZA was approved for the treatment of complicated urinary tract- and intra-abdominal infections by the US Food and Drug Administration (FDA) (Wang et al., 2020). In recent years, the resistance rate of CZA in *K. pneumoniae* has gradually increased during its widespread use in clinical practice (Jiang et al., 2022).

Recent studies have reported that the resistance mechanisms of CRKP against CZA mainly include mutations in KPC enzyme, outer membrane proteins OmpK35/36, porin LamB, and penicillin-binding proteins PBP2/3, or the variation of *bla*_KPC_ expression (Nelson et al., 2017; Shen et al., 2017; Giddins et al., 2018; Guo et al., 2021). Besides, overexpression of efflux pumps of AcrAB–TolC or MexAB–OprM efflux system was reported to be associated with CZA resistance in *K. pneumoniae* and *P. aeruginosa*, respectively (Xiong et al., 2022). Those studies have mainly focused on CZA resistance mechanisms and CZA heteroresistance in CRKP remains poorly understood. Heteroresistance, defined as the presence of a minority-resistant subpopulation with a higher minimum inhibitory concentration (MIC) than the majority-susceptible population, is a common phenotype in many bacteria (Stojowska-Swędrzyńska et al., 2021). Heteroresistance is difficult to detect and identify by standard antimicrobial susceptibility testing due to its phenotypic and genetic instability (Choby et al., 2021). Resistant subpopulations revert to the susceptible phenotype in the absence of selective pressure resulted from the fitness cost conferred by the resistance gene amplification or mutations (Nicoloff et al., 2019). Although the clinical relevance of antibiotic heteroresistance remain unclear, recent findings suggest that heteroresistance may result in antibiotic treatment failure following enrichment of the resistant subpopulations during antibiotic exposure (Andersson et al., 2019). Heteroresistance has been described for several antibiotics in *K. pneumoniae* (Zheng et al., 2018b; Abe et al., 2020; Zhang et al., 2021b; Moon et al., 2022; Sancak et al., 2022; Rajakani et al., 2023), while CZA heteroresistance in CRKP has yet to be systematically investigated.

The main purpose of this study was to uncover the distribution and potential mechanisms of CZA heteroresistance in CRKP isolates. The prevalence of CZA heteroresistant strains were examined by population analysis profiling (PAP) and their characteristics were detected by polymerase chain reaction (PCR). The underlying mechanism of CZA heteroresistance in CRKP strains was investigated by molecular sequencing, whole genome sequencing (WGS), quantitative real-time PCR (qRT-PCR), and *in vitro* functional experiments. In addition, strategies for preventing the emergence of CZA heteroresistance and alternative treatment options for strains exhibiting CZA heteroresistance were explored. These findings will inform the potential risks and corresponding therapeutic strategies for CZA heteroresistance in CRKP.

## 2 Materials and methods

### 2.1 Bacterial strains, media, and antibiotics

A total of 311 nonduplicate clinical CRKP isolates were collected from inpatients admitted to three tertiary hospitals in Guangdong province of China from 2020 – 2022. Clinical *K. pneumoniae* isolates were identified by standard methods using the VITEK 2 compact system (BioMérieux, Marcy l’Etoile, France). *K. pneumoniae* was grown at 37°C in Luria–Bertani (LB) broth (Oxoid Ltd., Basingstoke, UK), and Mueller-Hinton (MH) broth or agar (Oxoid, Basingstoke, UK). All procedures were performed in accordance with the ethical standards of Yangjiang People’s Hospital and the 1964 Helsinki Declaration and its later amendments. The antimicrobial agents used in this study were purchased from Meilunbio (Dalian, China), and *Escherichia coli* ATCC 25922 was used as the quality control strain.

### 2.2 Antimicrobial susceptibility testing

The MIC of CZA, other commonly used antibiotics (e.g., meropenem, imipenem, tigecycline, polymyxin B, minocycline, levofloxacin, ceftazidime, gentamicin, fosfomycin, ceftriaxone), and other combination therapies (e.g., ceftazidime-relebactam, aztreonam-avibactam, imipenem-relebactam, and meropenem-vaborbactam) were detected using the broth microdilution method according to Clinical and Laboratory Standards Institute (CLSI) guidelines (CLSI-M100-S34) (Clinical and Laboratory Standards Institute, 2024). MIC results of each antibiotic (except tigecycline) were interpreted based on the CLSI-recommended breakpoints. The susceptibility of tigecycline was interpreted according to the FDA breakpoints (Sheng et al., 2014). In this study, CRKP was confirmed using the modified Hodge test (Pourgholi et al., 2022), in accordance with the CLSI guidelines.

### 2.3 Population analysis profiles

PAP was used as a standard method to identify CZA heteroresistance among 275 CRKP isolates with a CZA MIC ≤8/4 mg/L as described previously (Nicoloff et al., 2019). In short, CZA-susceptible CRKP were cultured on blood agar plates for 24 h at 37°C. Cell suspensions were then prepared to match the 0.5 McFarland standard (1.0-1.5 × 10^8^ CFU/mL) and 50 μL aliquots were spread onto MH agar plates with or without CZA (0, 2/4, 4/4, 8/4, 16/4, 32/4, and 64/4 mg/L). The number of bacterial colonies were counted after overnight incubation at 37°C. According to the CZA resistance breakpoint of *K. pneumoniae*, CZA heteroresistance was defined as a CZA-susceptible isolate (MIC ≤8/4 mg/L) with subpopulations growing in the presence of CZA ≥16/4 mg/L at a detection threshold of 20 CFU/mL. Colonies selected from plates with a CZA concentration ≥16/4 mg/L were categorized as resistant subpopulations of each CZA heteroresistant isolate (name as: strain-RS). The parental strains of CZA heteroresistant isolates were considered as the susceptible subpopulation because dominant subpopulations are susceptible to CZA (name as: strain-HP). The CZA MICs of resistant subpopulations were reassessed after serial passaging on antibiotic-free medium to evaluate the stability of the heteroresistant phenotype (Li et al., 2024). The resistant subpopulations or their parental strains were cultured in MHB either supplemented with 4/4 mg/L CZA or without CZA, respectively, for the subsequent experiments.

### 2.4 Polymerase chain reaction and sequencing

Multilocus sequence typing (MLST), carbapenemase type, capsule serotype, and genetic mutations of CZA resistance genes were determined by PCR and sequencing. Total DNA was extracted using lysis buffer for microorganisms (Takara, Tokyo, Japan) from each CZA heteroresistant parental strain, as well as their resistant subpopulations. PCRs were performed using a PCR Mastermix (Thermo Fisher Scientific, Waltham, MA) according to the manufacturer’s instructions. DNA sequencing was performed using the Sanger method as previously described (Beyrouthy et al., 2017). MLST was performed based on the sequence analysis of fragments of seven housekeeping genes: *gapA, phoE, infB, pgi, rpoB, tonB* and *mdh*. The ST types were determined by the allelic combination of the seven genes on the Institute Pasteur’s MLST website (https://bigsdb.pasteur.fr/klebsiella/) (Perdigão et al., 2020). The carbapenemase type of *bla*_KPC_, *bla*_NDM_, *bla*_OXA-48_, *bla*_IMP_, and *bla*_VIM_ were identified as previously described (Lin et al., 2020b). The capsule serotypes were aligned with the *wzi* sequences deposited in the Institut Pasteur database(http://bigsdb.web.pasteur.fr) via *wzi* alleles (Hu et al., 2024). Genetic mutations in genes associated with CZA resistance to *K. pneumoniae*, including *bla*_KPC_, *ompK*35/36, *mdrA* (encoding PBP2)*, ftsI* (encoding PBP3), and *lamB*, were identified by comparing the sequence between the resistant subpopulations and their parental strains. All PCR primers are listed in **Supplementary Table S1**.

### 2.5 String test

The hypermucoviscosity phenotypes of *K. pneumoniae* isolates were confirmed by string test (Lin et al., 2020b). Bacterial strains were grown on a blood agar plate at 37°C overnight. A positive outcome in the string test was determined by the formation of a viscous string > 5 mm in length stretched by an inoculation loop.

### 2.6 Measurement of bacterial growth curves

Growth curves were measured in four groups of randomly selected resistant subpopulations and their parental strains (CRKP-46 HP&RS, CRKP-73 HP&RS, CRKP-271 HP&RS, and CRKP-301 HP&RS) with or without CZA pressure to assess potential fitness cost in CZA heteroresistance strains as described previously (Lin et al., 2020a). In brief, overnight bacterial cultures of resistant subpopulations and their parental strains were diluted 1:200 into 1 mL of MHB containing 4/4 mg/L CZA or into fresh MHB, respectively, of which 300 μL was added into each well of a 96-well plate. Three parallel wells were used for each sample. The plates were placed in a Bioscreen C MBR (Oy Growth Curves Ab Ltd., Helsinki, Finland), and the bacteria were grown at 37°C with shaking at 220 rpm. Growth curves of the strains were determined by measuring the optical density at 600nm (OD600) at 30-min intervals over a period of 24 h.

### 2.7 Whole-genome sequencing

WGS was then performed in five groups of randomly selected parental strain and their resistant subpopulation (CRKP-56 HP&RS, CRKP-62 HP&RS, CRKP-87 HP&RS, CRKP-179 HP&RS, and CRKP-277 HP&RS) to determine their genotypic changes as described previously (Guo et al., 2021). In brief, single colony from an overnight agar plate of each strains was cultured in 4 mL of LB broth at 37°C for 16 h, and genomic DNA was extracted using a Bacterial DNA Kit DP302 (Tiangen biotech, Beijing, China). WGS was performed by Novogene (Beijing, China) using an Illumina Hiseq™ X Ten platform (Illumina, San Diego, CA, USA). The raw data were trimmed and assembled by shovill (Trinetta et al., 2020). Prokka was used to annotate the assembled contigs (Seemann, 2014). Single nucleotide polymorphisms (SNPs) were identified using Snippy (Olawoye et al., 2020). Mutations in resistant subpopulations were identified by comparing the sequence with their parental strains.

### 2.8 Quantitative real-time PCR analysis

The relative expression and copy number of *bla*_KPC_, *ompK35*, *ompK36*, *lamB*, *acrA*, *and oqxA* were determined in resistant subpopulations and their parental strains using qRT-PCR as previously described (Zhang et al., 2020). For expression of these genes, overnight culture was inoculated 1:1,000 into 4 mL of fresh LB broth and cultured at 37°C shaking at 220 rpm until the growth reached the logarithmic phase. Total RNA was extracted using a Bacteria RNA Extraction Kit (Vazyme, Nanjing, China). Reverse transcription and qRT-PCR were performed using the HiScriptIII-RT SuperMix (Vazyme, Nanjing, China) and ChamQ Universal SYBR qRT-PCR Master Mix (Vazyme, Nanjing, China), respectively, in a LightCycler480II system (Roche, Basel, Switzerland). Threshold cycle (Ct) numbers were determined using the qRT-PCR system software, and the relative transcript levels were calculated using the 2^-ΔΔCT^ method with *rrsE* as the endogenous reference gene (Lin et al., 2020b). For copy number of these genes, total DNA was extracted using a TIANamp Bacteria DNA Kit (TIANGEN, Beijing, China), and qRT-PCR was performed as described above. Each CZA heteroresistant parental strain was used as the reference strain (expression level = 1, copy number = 1). The expression level and copy number of these genes in the resistant subpopulations were compared to those in the parental strains. All sample was run in triplicate. The primers used for qRT-PCR are listed in **Supplementary Table S2**.

### 2.9 Antisense RNA silencing

To verify the role of KPC expression in CZA heteroresistance in CRKP isolates, *bla*_KPC_ was silenced by antisense RNA (asRNA) in 7 groups of resistant subpopulations with high *bla*_KPC_ expression and their parental strains as previously described (Zheng et al., 2018a). First, the predicted Shine–Dalgarno sequence plus 138 nucleotides downstream of the start codon of *bla*_KPC_ was amplified. This segment was then digested with *Hin*dIII and *Bam*HI endonucleases and inserted into the isopropyl β-D-thiogalactoside (IPTG) - inducible asRNA-expressing plasmid, pHN678 (Nakashima et al., 2006). The constructed plasmid, pASKPC, was confirmed by PCR and DNA sequencing, and transformed separately into the resistant subpopulations or their parental strains by electroporation, then verified again using PCR. The pASKPC plasmid was also introduced in 3 groups of resistant subpopulations without high *bla*_KPC_ expression and their parental strains as a control. Simultaneously, the pHN678 plasmid was introduced into the same isolates as a vector control. The silencing efficacy of *bla*_KPC_ in silenced strains was measured by qRT-PCR as described above. Bacteria were pre-treated with 1 mM IPTG to induce expression of the asRNA silencing plasmids. The CZA MIC of *bla*_KPC_-silenced resistance subpopulations was assessed using the broth microdilution method. The formation of CZA heteroresistance in the *bla*_KPC_-silenced parental strains was determined using the PAP test. All strains and primers used for asRNA silencing are listed in **Supplementary Tables S3, S4**.

### 2.10 Inhibition strategies of heteroresistance

The inhibition effect of different avibactam concentrations on CZA MIC in resistant subpopulations and the emergence of CZA heteroresistance in susceptible CRKP strains were examined. After increasing the concentrations of avibactam to 8 or 16 mg/L, CZA MICs were determined in resistant subpopulations using the broth microdilution method and CZA heteroresistance in 275 CZA-susceptible CRKP strains was determined using the PAP assay as described above. Additionally, combination therapy-oriented prevention of CZA heteroresistance was evaluated in susceptible CRKP strains using the PAP test with the addition of tigecycline, polymyxin B, levofloxacin, or fosfomycin to the MH agar plates at a concentration of 0.25 × or 0.5 × MIC.

### 2.11 Statistical analysis

Statistical significance of the differences in expression levels and copy numbers of CZA resistance genes between the resistant subpopulations and their parental strains was analyzed by Student’s *t*-test. The chi-square test was used to analyze the incidence of CZA heteroresistance in CRKP isolates after following combination use with other antibiotics. A repeated measures analysis of variance (ANOVA) was used to assess changes in bacterial growth. The statistical analyses were performed using the IBM SPSS Statistics (version 22.0; IBM, Chicago, USA) and GraphPad Prism software (version 8.43; GraphPad Software, San Diego, CA, USA). *P*-values <0.05 were regarded as statistically significant.

### 2.12 Genome accession numbers

The genome sequencing data were deposited in the NCBI database under BioProject no. PRJNA1164017 (https://www.ncbi.nlm.nih.gov/bioproject/PRJNA1164017/).

## 3 Results

### 3.1 Frequency of ceftazidime-avibactam heteroresistance in clinical CRKP isolates

The 311 CRKP clinical strains were isolated from various infective sample sources, including sputum (41.8%), blood (25.4%), urine (18.6%), and others (14.2%) (**Supplementary Figure S1**). Moreover, the susceptibilities of those isolates to CZA and other antibiotics are summarized in **Table 1**. The resistance rate of CZA in these 311 CRKP strains was 11.6% with MIC range for 0.125/4–128/4 mg/L, MIC_50_ and MIC_90_ for 2/4 mg/L and 16/4 mg/L, respectively. The CZA heteroresistance of 275 CRKP isolates with CZA MIC ≤ 8/4 mg/L was determined using the PAP test. A total of 34 CRKP strains (12.4%) were identified to be heteroresistant to CZA. Furthermore, the incidence of CZA heteroresistance in CRKP isolates was increased along with the CZA MIC (**Table 2**).

**Table 1:** Susceptibility of CZA and other commonly used antibiotics for 311 CRKP clinical isolates.

Antibiotic	MIC (mg/L)			No. (%) of isolates and their susceptibility category		
	Range	MIC_50_	MIC_90_	S^a^	I^b^	R
Ceftazidime-Avibactam	0.125/4–128/4	2/4	16/4	275 (88.4)	/	36 (11.6)
Tigecycline	0.25–32	1	2	288 (92.6)	12 (3.9)	11 (3.5)
Polymyxin B	0.125–16	0.5	2	288 (92.6)	/	23 (7.4)
Minocycline	0.5–128	8	16	121 (38.9)	65 (20.9)	125 (40.2)
Meropenem	16–>128	128	>128	0 (0)	0 (0)	311 (100.0)
Imipenem	8–>128	32	64	0 (0)	0 (0)	311 (100.0)
Levofloxacin	1–>128	16	32	0 (0)	3 (1.0)	308 (99.0)
Ceftazidime	16–>128	64	>128	0 (0)	0 (0)	311 (100.0)
Gentamicin	0.5–>128	32	64	27 (8.7)	0 (0)	284 (91.3)
Ceftriaxone	32–>128	64	>128	0 (0)	0 (0)	311 (100.0)

a MIC results of each antibiotic (except tigecycline) were interpreted based on the CLSI-recommended breakpoints. The susceptibility of tigecycline was interpreted according to the FDA breakpoints. b“/”: the antibiotic has no intermediate breakpoints.

**Table 2:** Distribution of CZA heteroresistance among CZA-susceptible CRKP isolates.

Distribution of CZA MIC (mg/L)	PAP test^a^		Heteroresistant rate (%)
	Positive (n)	Negative (n)	
MIC ≤1/4 (47^b^)	0	47	0
MIC =2/4 (112)	4	108	3.6
MIC =4/4 (92)	16	76	17.4
MIC =8/4 (24)	14	10	58.3
Total (275)	34	241	12.4

a CZA heteroresistance is defined as CZA-susceptible isolates (CZA MIC of ≤ 8/4 mg/L) with subpopulations growing in the presence of ≥ 16/4 mg/L CZA, with a detection limit of 20 CFU/mL.

b Number of strains with different MIC values.

The resistant subpopulations of each CZA heteroresistant isolate were selected from the plates with ≥16/4 mg/L CZA concentrations in the PAP test. The parental strains of CZA heteroresistant isolates were classified as susceptible subpopulations in this study because the majority of subpopulations were susceptible to CZA. The CZA MIC of resistant subpopulations was 4- to 16-fold higher than that of the parental strains (**Table 3**). The stability of the resistant subpopulations was analyzed by serial passaging on culture medium without antibiotics. The CZA MIC of resistant subpopulations reversed to susceptible levels after 10 passages on antibiotic-free medium, and subsequently returned to the MIC level of parental strains after 30 passages (**Supplementary Table S5**). These findings suggest that the resistant subpopulations of CZA heteroresistant isolates are unstable and could restore sensitivity to CZA after removal of the antibiotic pressure.

**Table 3:** Characteristics of the CZA heteroresistant CRKP isolates.

Strain	CZA MIC (mg/L)		Source	MLST	Carbapenemase types	Capsular types	String-test^c^
	HP^a^	RS					
CRKP-13	4/4	16/4	Urine	11	KPC-2	KL47	–
CRKP-29	2/4	16/4	Blood	11	KPC-2	KL47	+
CRKP-40	4/4	16/4	Sputum	11	KPC-2	KL64	–
CRKP-46	4/4	16/4	Urine	11	KPC-2	KL64	–
CRKP-56	2/4	16/4	Abscess fluid	11	KPC-2	KL47	+
CRKP-60	8/4	32/4	Sputum	11	KPC-2	KL64	–
CRKP-62	4/4	16/4	Blood	11	KPC-2	KL47	–
CRKP-73	8/4	32/4	Blood	37	KPC-2	KL12	–
CRKP-87	4/4	32/4	Sputum	11	KPC-3	KL64	–
CRKP-91	4/4	16/4	Ascites	NT^b^	KPC-2	KL47	–
CRKP-96	4/4	16/4	Blood	11	KPC-2	KL64	–
CRKP-105	2/4	32/4	Blood	15	KPC-2	KL19	–
CRKP-106	4/4	16/4	Sputum	11	KPC-2	KL47	+
CRKP-119	8/4	32/4	Blood	15	KPC-2	KL19	–
CRKP-122	8/4	32/4	Sputum	11	KPC-2	KL64	–
CRKP-135	4/4	16/4	Blood	65	KPC-2	KL2	+
CRKP-141	8/4	64/4	Wound secretion	11	KPC-2	KL64	–
CRKP-153	8/4	32/4	Urine	1296	KPC-2	KL75	–
CRKP-161	8/4	32/4	Sputum	11	KPC-2	KL47	–
CRKP-168	4/4	16/4	Sputum	11	KPC-2	KL47	–
CRKP-179	8/4	32/4	Wound secretion	11	KPC-2	KL64	–
CRKP-184	4/4	16/4	Sputum	11	KPC-2	KL47	–
CRKP-196	8/4	32/4	Urine	23	KPC-2	KL1	+
CRKP-209	8/4	64/4	Sputum	15	KPC-2	KL19	–
CRKP-213	4/4	32/4	Ascites	37	KPC-2	KL14	–
CRKP-236	4/4	16/4	Drainage	45	KPC-2	KL24	–
CRKP-251	4/4	16/4	Cerebrospinal fluid	11	KPC-2	KL47	–
CRKP-255	2/4	32/4	Cerebrospinal fluid	11	KPC-2	KL64	–
CRKP-259	4/4	16/4	Blood	11	KPC-2	KL47	+
CRKP-265	8/4	32/4	Urine	37	KPC-2	KL14	–
CRKP-271	4/4	16/4	Bile	11	KPC-2	KL47	–
CRKP-277	8/4	32/4	Sputum	11	KPC-2	KL64	–
CRKP-286	8/4	64/4	Sputum	NT	KPC-2	KL47	–
CRKP-301	8/4	32/4	Sputum	11	KPC-2	KL64	–

a HP: CZA heteroresistant parental strain; RS: resistant subpopulation of CZA heteroresistant strain.

b NT: not typeable.

c +: positive; –: negative.

### 3.2 Characteristics of ceftazidime-avibactam heteroresistance in CRKP isolates

The source, MLST, carbapenemase type, capsule serotype, and hypermucoviscosity phenotype of CZA heteroresistant isolates are summarized in **Table 3**. Results showed that the heteroresistant strains came from diverse specimens, including sputum (n = 12, 35.3%), blood (n = 8, 23.5%), urine (n = 5, 14.7%), and others (n = 9, 26.5%). MLST revealed seven STs in 34 CZA heteroresistant strains, with ST11 being the dominant type (n = 22, 64.7%). The carbapenemase type of all heteroresistant isolates belonged to KPCs (KPC-2, 97.1%; KPC-3, 2.9%). Among 34 CZA heteroresistant isolates, KL47 (n = 13, 38.2%), and KL64 (n = 11, 32.4%) were the prevalent capsular serotypes. Notably, six strains were identified with a hypermucoviscosity phenotype by string test.

To investigate the cross-resistance among CZA, other commonly use antibiotics and other combination agents, *in vitro* antimicrobial activity against resistant subpopulations and parental strains were compared using the broth microdilution method. The MICs of tigecycline, polymyxin B, meropenem, levofloxacin, gentamicin, and ceftriaxone showed no significant differences between resistant subpopulations and parental strains. Both strains exhibited high resistance to meropenem, levofloxacin, gentamicin, and ceftriaxone, but remained sensitive to tigecycline and polymyxin B. Ceftazidime-relebactam and aztreonam-avibactam showed a 4-fold MIC increase in 70.6% (24/34) and 58.8% (20/34) of the resistant subpopulations compared to their parental strains, respectively. It indicates a potential cross-resistance between these combination drugs and CZA. Although imipenem-relebactam and meropenem-vaborbactam also showed a 4-fold MIC increase in 29.4% (10/34) and 38.2% (13/34) of the resistant subpopulations, respectively, both drugs still demonstrated excellent antimicrobial activity against the resistant subpopulations (**Table 4**).

**Table 4:** Comparison of the antimicrobial susceptibilities of CZA heteroresistant parental strains and their resistant subpopulations to other antibiotics.

Antibiotics	MIC of HP^a^ strains (mg/L) (n = 34)				MIC of RS strains (mg/L) (n = 34)				Fold change ≥4^b^ (n, %)
	Range	MIC_50_	MIC_90_	Num. of Resistance (%)	Range	MIC_50_	MIC_90_	Num. of Resistance (%)	
Tigecycline	0.5–8	1	2	2 (5.9)	0.5–8	1	2	2 (5.9)	0 (0)
Polymyxin B	0.5–16	1	2	3 (8.8)	0.5–16	1	2	3 (8.8)	0 (0)
Meropenem	64–>128	128	>128	34 (100)	64–>128	128	>128	34 (100)	0 (0)
Levofloxacin	8–128	32	64	34 (100)	8–128	32	64	34 (100)	0 (0)
Gentamicin	2–>128	64	128	33 (97.0)	2–>128	64	128	33 (97.0)	0 (0)
Ceftriaxone	64–>128	128	>128	34 (100)	64–>128	128	>128	34 (100)	0 (0)
Ceftazidime-relebactam	16/4–128/4	64/4	128/4	/^c^	32/4–>512/4	256/4	512/4	/	24 (70.6)
Aztreonam-avibactam	1/4– 8/4	4/4	8/4	/	2/4–>128/4	16/4	32/4	/	20 (58.8)
Imipenem-relebactam	0.12/4– 4/4	0.25/4	0.5/4	1 (2.9)	0.12/4– 8/4	0.5/4	2/4	3 (8.8)	10 (29.4)
Meropenem-vaborbactam	0.12/8– 16/8	0.5/8	1/8	2 (5.9)	0.25/8–>32/8	1/8	16/8	4 (11.8)	13 (38.2)

a HP: heteroresistant parental strains; RS: resistant subpopulations of CZA heteroresistant isolates.

b Number of resistant subpopulations with MIC values ≥4 times higher than heteroresistant parental strains.

c ”/”: the resistance breakpoints are not available.

To assess the impact of the emergence of CZA heteroresistance on the growth capacity of these strains, growth curves were detected in four groups of randomly selected resistant subpopulations and their parental strains with or without CZA pressure. Results showed that 75% (3/4) of resistant subpopulations exhibited a significant decrease in growth capacity under 4/4mg/L CZA pressure compared to their parental strains. However, there was no significant difference in growth capacity between resistant subpopulations and their parental strains under antibiotic-free conditions (**Supplementary Figure S2**). These results suggest that the resistant subpopulations may incur a fitness cost under antibiotic pressure and lead to attenuated growth capacity.

### 3.3 Ceftazidime-avibactam heteroresistance mechanism in CRKP isolates

To investigate the mechanisms of CZA heteroresistance, mutations in CZA resistance-associated genes, including KPC, OmpK35/36, PBP2/3, and LamB, were identified in all resistant subpopulations by PCR and sequencing. The results showed that one strain harbored mutant KPC (G142D), one strain had a double point mutation (V152A, K264R) in OmpK35, and two strains had mutations in OmpK36 (D199G and N229S, respectively), while no mutation was detected in PBP2/3 and LamB (**Table 5**). The above mutations are relatively scattered without significant regularity, suggesting that mutations in these genes are not responsible for the formation of CZA heteroresistance. WGS was performed on five groups of randomly selected parental strains and their resistant subpopulations to determine if there were any additional resistance genes contributing to CZA heteroresistance. Results showed that 80% (4/5) of the resistant subpopulations had an I5V mutation in RffH, 60% (3/5) of the resistant subpopulations harbored a D48E mutation in RmlB, one strain carried a VK688AE mutation in RsxC, and one strain had an I248L mutation in DgcP. No mutation in resistance genes was identified in any of the five resistant subpopulations (**Supplementary Table S6**). These results indicate that genetic mutation in resistance genes is not the main reason for CZA heteroresistance in CRKP isolates.

**Table 5:** Mutations of KPC, OmpK35/36, PBP2/3 and LamB in resistant subpopulations of CZA heteroresistant strains.

Resistant subpopulations	Mutations					
	KPC^a^	OmpK35	OmpK36	PBP2	PBP3	LamB
CRKP-13-RS^b^	W^c^	W	W	W	W	W
CRKP-29-RS	W	W	W	W	W	W
CRKP-40-RS	W	W	W	W	W	W
CRKP-46-RS	W	W	D199G	W	W	–^d^
CRKP-56-RS	W	W	W	W	W	W
CRKP-60-RS	G142D	W	W	W	W	W
CRKP-62-RS	W	W	W	W	W	W
CRKP-73-RS	W	W	W	W	W	–
CRKP-87-RS	W	W	W	W	W	W
CRKP-91-RS	W	V152A, K264R	W	W	W	W
CRKP-96-RS	W	W	W	W	W	W
CRKP-105-RS	W	W	W	W	W	W
CRKP-106-RS	W	W	W	W	W	–
CRKP-119-RS	W	W	W	W	W	W
CRKP-122-RS	W	W	W	W	W	W
CRKP-135-RS	W	W	W	W	W	W
CRKP-141-RS	W	W	–	W	W	W
CRKP-153-RS	W	W	W	W	W	W
CRKP-161-RS	W	W	W	W	W	W
CRKP-168-RS	W	W	W	W	W	W
CRKP-179-RS	W	W	W	W	W	W
CRKP-184-RS	W	W	W	W	W	W
CRKP-196-RS	W	W	N229S	W	W	W
CRKP-209-RS	W	W	W	W	W	W
CRKP-213-RS	W	W	W	W	W	W
CRKP-236-RS	W	W	W	W	W	W
CRKP-251-RS	W	W	W	W	W	W
CRKP-255-RS	W	W	W	W	W	W
CRKP-259-RS	W	W	W	W	W	W
CRKP-265-RS	W	W	W	W	W	W
CRKP-271-RS	W	W	W	W	W	W
CRKP-277-RS	W	W	W	W	W	W
CRKP-286-RS	W	W	W	W	W	W
CRKP-301-RS	W	W	W	W	W	W

a Mutations were determined by comparing the sequence between the resistant subpopulations and their parental strains.

b RS, resistant subpopulations of CZA heteroresistant isolates.

c W, no mutation was observed in the resistant subpopulations compared with the parental strains.

d ”–”The gene was not detected in the strains.

**Figure 1:**
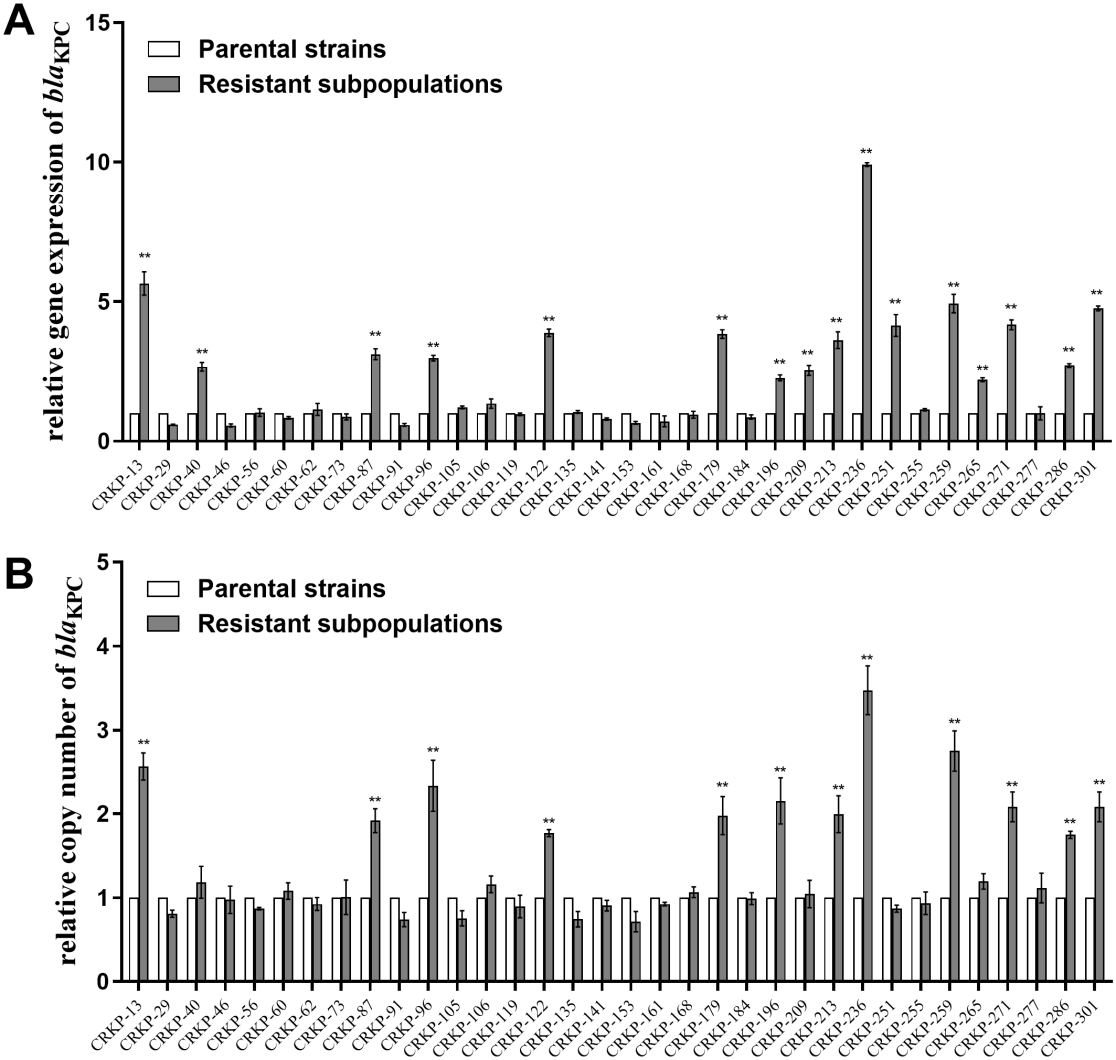
Relative *bla*_KPC_ expression level and copy number in resistant subpopulations compared with their parental strains. The parental strains and resistant subpopulations were grown in LB to the logarithmic phase for RNA extraction and to the stationary phase for DNA extraction. Total RNA or total DNA were extracted. The expression level **(A)** and copy number **(B)** of *bla*_KPC_ were then examined by qRT-PCR. The housekeeping gene, *rrsE*, was used as the endogenous reference gene. The parental strains were used as the reference strain (expression level = 1, copy number = 1). All experiments were carried out in triplicate. **P<0.05.

The relative expression and copy number of *bla*_KPC_, *ompK35*, *ompK36*, *lamB*, *acrA*, *and oqxA* were then compared between CZA heteroresistant parental strains and their resistant subpopulations. The expression of *bla*_KPC_ was significantly upregulated in 47.1% (16/34) of the resistant subpopulations compared to the parental strains (**Figure 1A**). Moreover, the copy number of *bla*_KPC_ was significantly higher in 35.3% (12/34) of resistant subpopulations compared to the parental strains (**Figure 1B**). No significant differences were found in the expression levels and copy numbers of other genes (**Supplementary Figure S3**). These results suggest that high expression of *bla*_KPC_ may be associated with CZA heteroresistance.

### 3.4 Validation of the roles of KPC expression in ceftazidime-avibactam heteroresistance

To further confirm the relationship between KPC expression and CZA heteroresistance in CRKP isolates, asRNA technology was used to silence *bla*_KPC_ expression in resistant subpopulations and their parental strains. The asRNA plasmid, pASKPC, was constructed and transformed into seven resistant subpopulations with high expression of *bla*_KPC_ and three resistant subpopulations without high expression of *bla*_KPC_, respectively. The pASKPC plasmid was also transformed into their parental strains to assess the formation of CZA heteroresistance. The silencing efficacy of *bla*_KPC_ was firstly confirmed by qRT-PCR in the constructed strains. The expression of *bla*_KPC_ was downregulated by over 80% in the transformed strains compared to the wild-type strains following IPTG induction (**Supplementary Figure S4**). The effect of *bla*_KPC_ silencing on CZA heteroresistance was then determined by antimicrobial susceptibility testing and PAP assays. For resistant subpopulations with high *bla*_KPC_ expression, the CZA MICs decreased by 4- to 8-fold after silencing *bla*_KPC_ and returned to susceptible levels. Their parental strains lost the CZA heteroresistant phenotype after transforming pASKPC. For resistant subpopulations without high *bla*_KPC_ expression, transformed with pASKPC did not affect the CZA MIC in resistant subpopulations and the formation of CZA heteroresistance in their parental strains (**Table 6**; **Supplementary Table S7**). These results suggest that high expression of KPC contributes to the formation of CZA heteroresistance in CRKP isolates.

**Table 6:** CZA susceptibility of *bla*_KPC_-silenced resistance subpopulations.

Transformed plasmid	Strain	CZA MIC (mg/L)^a^			
		Wild-type strain^b^	Vector control strain^c^	Derivative strain^d^	Parental strain^e^
KPC overexpression strains					
pASKPC	CRKP-13-RS	16/4	16/4	4/4	4/4
	CRKP-122-RS	32/4	32/4	8/4	8/4
	CRKP-179-RS	32/4	16/4	4/4	8/4
	CRKP-236-RS	16/4	8/4	2/4	4/4
	CRKP-251-RS	16/4	16/4	4/4	4/4
	CRKP-259-RS	16/4	16/4	4/4	4/4
	CRKP-301-RS	32/4	16/4	4/4	8/4
Non KPC overexpression strains					
pASKPC	CRKP-29-RS	16/4	16/4	8/4	2/4
	CRKP-73-RS	32/4	32/4	16/4	8/4
	CRKP-277-RS	32/4	16/4	16/4	8/4

a CZA MIC was detected in strains after induction with 1.0 mM IPTG.

b Wild-type strain: Resistant subpopulation of CZA heteroresistant strain.

c Vector control strain: wild-type strain transformed with pHN678.

d Derivative strain: wild-type strain transformed with pASKPC.

e Parental strain: wild-type CZA heteroresistant parental strain.

### 3.5 Inhibition strategies for the formation of CZA heteroresistance in CRKP isolates

The results described above suggested that overexpression of KPC may lead to CZA heteroresistance. Avibactam directly binds the KPC enzyme to inhibit its function, suggesting that increasing avibactam concentrations may prevent the emergence of CZA heteroresistance. Next, we investigated the inhibitory effect of increasing avibactam concentrations on the CZA MIC of resistant subpopulations and the formation of CZA heteroresistance in susceptible CRKP strains. The CZA MIC was significantly decreased by ≥ 4-fold in 76.5% (26/34) and in 97.0% (33/34) of the resistant subpopulations following with avibactam concentrations increased to 8 or 16 mg/L, respectively (**Supplementary Table S8**). These data suggested that increasing avibactam concentrations could significantly decrease the CZA MIC of resistant subpopulations. In addition, PAP test data revealed that only three CZA heteroresistant strains were detected from 275 CRKP isolates after increasing the avibactam concentration to 8 mg/L, and only one CZA heteroresistant strain was identified at 16 mg/L avibactam, significantly fewer than those detected at avibactam concentration of 4 mg/L (**Table 7**). These results indicated that increasing the concentration of avibactam could be an effective strategy for preventing the development of CZA heteroresistance in CRKP isolates.

**Table 7:** Distribution of CZA heteroresistance of clinical CRKP isolates in different avibactam concentration or combining with other antibiotics.

Distribution of CZA MIC (mg/L)	Positive results of PAP test for CZA heteroresistance (n, %)^a^										
	Concentration of avibactam			+ tigecycline^b^		+ polymyxin B		+ levofloxacin		+ fosfomycin	
	4 mg/L	8 mg/L	16 mg/L	0.25×	0.5×	0.25×	0.5×	0.25×	0.5×	0.25×	0.5×
MIC ≤1/4 (47^c^)	0 (0)	0 (0)	0 (0)	0 (0)	0 (0)	0 (0)	0 (0)	0 (0)	0 (0)	0 (0)	0 (0)
MIC =2/4 (112)	4 (3.6)	0 (0)	0 (0)	0 (0)	0 (0)	2 (1.8)	1 (0.9)	3 (2.7)	3 (2.7)	3 (2.7)	2 (1.8)
MIC =4/4 (92)	16 (17.4)	1(1.9)	0 (0)	10 (10.9)	8 (8.7)	15 (16.3)	12 (13.0)	16 (17.4)	15 (16.3)	15 (16.3)	13 (14.1)
MIC =8/4 (24)	14 (58.3)	2 (8.3)	1 (4.2)	8 (33.3)	4 (16.7)	14 (58.3)	14 (58.3)	14 (58.3)	14 (58.3)	14 (58.3)	13 (54.2)
Total (275)	34 (12.4)	3 (1.1)	1 (0.4)	18 (6.5)	12 (4.4)	31 (11.3)	27 (9.8)	33 (12.0)	32 (11.6)	32 (11.6)	28 (10.2)

a CZA heteroresistance was identified by PAP assay with the concentration of avibactam increased to 8mg/L or 16mg/L, or combining with other antibiotics. CZA heteroresistance is defined as an CZA-susceptible isolates (CZA MIC of ≤ 8/4 mg/L) with subpopulations growing in the presence of ≥ 16/4 mg/L CZA, with a detection limit of 20 CFU/mL. For avibactam concentrations of 8 or 16 mg/L, CZA heteroresistance is defined as an CZA-susceptible isolates (CZA MIC of ≤ 8/4 mg/L) with subpopulations growing in the presence of ≥ 16/8 mg/L or ≥ 16/16 mg/L CZA, respectively.

b + tigecycline: The combination of CZA and tigecycline, PAP test was performed after adding tigecycline to MH agar plates at the concentration of 0.25× or 0.5× MIC, respectively. The other combinations shared the same principle.

c Number of strains with different MIC values.

The inhibitory effect on CZA heteroresistance by the combination with other antibiotics were then explored. The incidence of CZA heteroresistance in CRKP isolates was examined by PAP test with separately adding other antibiotics. We found that the CZA heteroresistance rate decreased to 6.5% (18/275) following the addition of 0.25 × MIC tigecycline, and decreased to 4.4% (12/275) after adding 0.5 × MIC tigecycline, significantly lower than that detected without adding tigecycline (6.5% vs. 12.4%, *P*=0.02; 4.4% vs. 12.4%, *P*=0.001). The CZA heteroresistance rate showed no significantly decrease by the combination with polymyxin B, levofloxacin or fosfomycin (**Table 7**). These results indicated that combination with tigecycline could partly suppress CZA heteroresistance in CRKP isolates.

## 4 Discussion

CRKP is one of the most common pathogens responsible for nosocomial infections, including pneumonia, urinary tract infections, and bacteremia (Stojowska-Swędrzyńska et al., 2021). CRKP infections increase the mortality of severely ill hospitalized patients and elevate the financial burden on health systems globally (Karampatakis et al., 2023b). CRKP is resistant to almost all commonly used antibiotics, and clinically available agents are now limited (Karampatakis et al., 2023a). CZA is one of the most effective antibiotics for the treatment of CRKP infections and confers excellent antimicrobial activity against CRKP with the exception of strain that produce metallo-β lactamases (Ehmann et al., 2013). The emergence of CZA resistance in CRKP has resulted in limited effective treatment strategies and poses a significant challenge to public health security. Previous studies have focused on the mechanisms of CZA resistance, while CZA heteroresistance in CRKP remains poorly understood. This study sought to investigate the characteristics and mechanisms of CZA heteroresistance in CRKP isolates to inform appropriate treatment options for clinical CRKP infections.

In this study, CZA resistance rate was 11.6%, with MIC_50_ and MIC_90_ for 2/4 mg/L and 16/4 mg/L, respectively, which were similar to previous studies in China (Yin et al., 2019). Moreover, 12.4% of the CZA-susceptible CRKP isolates were found to exhibit heteroresistance to CZA. Previous studies have shown that *K. pneumoniae* has developed heteroresistance to a variety of antibiotics, including carbapenems, polymyxins, tetracyclines, aminoglycosides, fosfomycin, and cefiderocol (Zheng et al., 2018b; Abe et al., 2020; Zhang et al., 2021b; Moon et al., 2022; Sancak et al., 2022; Rajakani et al., 2023). Our data suggest that *K. pneumoniae* could also develop heteroresistance to compound preparations containing β-lactams-β-lactamase inhibitor combinations. As resistant subpopulations of heteroresistance occurs at a low frequency, it is difficult to detect heteroresistance in clinical isolates using conventional antibiotic susceptibility testing methods. Consequently, heteroresistant strains are frequently misclassified as susceptible strains (Band and Weiss, 2019). Therefore, heteroresistance is usually associated with an increased risk of recurrent infections and antibiotic treatment failure (Stojowska-Swędrzyńska et al., 2021). Accurate monitoring of the occurrence of CZA heteroresistance is essential for providing information about the potential risk of CZA resistance in clinical CRKP strains. Notably, we found that the frequency of CZA heteroresistance in CRKP strains increased along with the CZA MIC. This finding indicated that the incidence of CZA heteroresistance in CRKP may be MIC dependent, which is consistent with previous reports (Kim et al., 2020; Jo and Ko, 2021; Li et al., 2024). Strains with CZA MICs close to the resistance breakpoint were more likely to develop heteroresistance. Thus, it is essential to monitor strains with MICs near the CZA resistance breakpoint in the clinical detection, and appropriate antibiotic therapy strategies should be developed to reduce the risk of heteroresistance. The CZA MIC of resistant subpopulations reverted to susceptible levels after serial passaging on antibiotic-free medium. It indicates that CZA heteroresistance in CRKP strains is unstable and reversible, which is similar to the characteristics of heteroresistance to other antibiotics in CRKP reported previously (Andersson et al., 2019; Sato et al., 2020; Zhang et al., 2021a). 75% (3/4) of resistant subpopulations exhibited a significant decrease in growth capacity under CZA pressure, suggesting that the emergence of CZA heteroresistance may be accompanied by fitness costs and lead to an attenuated growth capacity.

In the current study, CZA heteroresistant strains mainly belonged to ST11, a dominant clone of CRKP in China (Liao et al., 2020). All of the heteroresistant strains produced KPC enzymes (KPC-2 or KPC-3), suggesting that the emergence of CZA heteroresistance may be related to the expression of KPC. CZA is particularly useful for treating KPC-producing *K. pneumoniae* infections, with an overall excellent activity (Findlay et al., 2021). The emergence of CZA heteroresistance in KPC-producing *K. pneumoniae* should be attracted highly attention in clinical practice, and control strategies should be taken to reduce the risk of its emergence and transmission. Notably, six CZA heteroresistant stains were identified with a hypermucoviscosity phenotype, suggesting that these strains may be hypervirulent *K. pneumoniae* strains (Li et al., 2023). The emergence of carbapenem-resistant hypervirulent *K. pneumoniae* with CZA heteroresistance is alarming. These strains simultaneously exhibited multidrug resistance, hypervirulence, and high transmissibility, making it more difficult to treat such infections clinically (Pu et al., 2023). Therefore, it is important to monitor the prevalence of heteroresistant phenotypes in hypervirulent CRKP to avoid outbreaks of these infections and the dilemma of no treatment options available. Additionally, resistant subpopulations exhibit significant cross-resistance to ceftazidime-relebactam and aztreonam-avibactam, indicating a trend towards cross-resistance with combination drugs containing either ceftazidime or avibactam as a component. Although a minority of resistant subpopulations showed an increase in MICs for imipenem-relebactam and meropenem-vaborbactam, both combinations still demonstrated excellent antimicrobial activity against resistant subpopulations. These results suggest that imipenem-relebactam and meropenem-vaborbactam may serve as alternative therapeutic options when clinical isolates with CZA heteroresistance are detected. Moreover, previous studies have shown that KPC mutation-mediated CZA resistance was generally accompanied by a substantial decrease in carbapenem MICs or even restoration of carbapenem susceptibility (Shields et al., 2017; Liao et al., 2022). However, all of the resistant subpopulations still showed high resistance to meropenem in this study, suggesting that CZA heteroresistance may not be mediated by KPC mutation.

Mutations in KPC, OmpK35/36, PBP2/3, and LamB, or the differential expression of KPC have been reported to be responsible for CZA resistance in CRKP (Nelson et al., 2017; Shen et al., 2017; Giddins et al., 2018; Guo et al., 2021). To investigate whether these mechanisms are associated with CZA heteroresistance, genetic mutations were examined in all resistant subpopulations by PCR and sequencing. The results showed that a few mutations in KPC and OmpK35/36 were detected in resistant subpopulations, but these mutations did not show significant regularity, suggesting that mutations in these CZA resistance genes may not be the main factor mediating the formation of CZA heteroresistance. Moreover, genotypic changes were detected in five groups of randomly selected parental strain and their resistant subpopulation by WGS. Mutations in RffH and RmlB were detected in 80% (4/5) and 60% (3/5) of CZA resistant subpopulations, respectively. RffH and RmlB are both involved in the biosynthesis of dTDP-rhamnose, which is essential for the growth of various bacteria (Li et al., 2006; van der Beek et al., 2019; Rao and Kuzminov, 2020). It suggests that mutations in these proteins may be associated with the decreased growth capacity of CZA heteroresistance strains. No significant mutations in the resistance genes were identified in the above experiments, indicating that mutations in resistance genes may not be responsible for the development of CZA heteroresistance. The expression levels and copy numbers of *bla*_KPC_, *ompK35*, *ompK36*, *lamB*, *acrAB*, *and oqxAB* were then compared between resistant subpopulations and their parental strains using qRT-PCR. Results showed that the relative expression and copy number of *bla*_KPC_ were significantly upregulated in 47.1% and 35.3% of the resistant subpopulations compared with their parental strains, respectively. It indicates that the high expression level and increased copy number of *bla*_KPC_ may contribute to the formation of CZA heteroresistance. Moreover, most resistant subpopulations with high *bla*_KPC_ expression were accompanied by an increase in *bla*_KPC_ copy number (**Supplementary Table S9**). These data suggest that the high expression of *bla*_KPC_ in resistant subpopulations may be caused by an increased copy number of *bla*_KPC_-carrying plasmids, which further leads to an increased fitness cost in the bacterial and a decline in growth capacity.

To confirm the role of KPC expression in CZA heteroresistant isolates, asRNA technology was used to silence *bla*_KPC_ in resistant subpopulations and their parental strains. Results showed that silencing *bla*_KPC_ significantly reduced the CZA MIC in resistant subpopulations with high *bla*_KPC_ expression and prevented the development of CZA heteroresistance in their parental strains. These data suggest that high expression of KPC contributes to CZA heteroresistance in CRKP isolates. KPC enzymes threaten the use of all current β-lactam antibiotics due to their hydrolysis reactions (Papp-Wallace et al., 2010). Avibactam inactivates KPC by covalently bonding to its active site, which inhibits the hydrolysis of ceftazidime, thereby protecting and enhancing the antibacterial activity of ceftazidime (Sy et al., 2019). High expression of KPC may weaken the inactivation effect of avibactam, further leading to the formation of CZA heteroresistance. Zhang et al. found that the excessive expression of KPC contributed to low-level CZA resistance in clinical CRKP isolates (Zhang et al., 2020). The MIC distribution and resistance mechanism of the low-level CZA resistant isolates are similar to the resistant subpopulations of CZA heteroresistance strains in our study. It suggests that low-level CZA resistant isolates may be developed from resistant subpopulations of CZA heteroresistant strains due to the use of CZA in clinical practice. The selection of antibiotic-resistant isolates may occur during discontinuous exposure to subtherapeutic drug concentrations, producing initially heteroresistant and finally fully resistant bacterial populations (Zhang et al., 2014). This requires accurate and consistent monitoring for early detection of emergent heteroresistance in the subtherapeutic drug concentration during the clinical use of CZA. Meanwhile, the exploitation of inhibition strategies to prevent CZA heteroresistance is critical in clinical settings.

Our data shows that the emergence of CZA heteroresistance may be due to the overexpression of KPC, leading to incomplete inactivation of KPC enzymes by avibactam. Therefore, the effect of increasing the concentration of avibactam on CZA heteroresistance was examined. The results showed that the CZA MIC of resistant subpopulations significantly decreased with increasing concentrations of avibactam. This effect was more pronounced when further increased avibactam concentration. Moreover, 97% of the parental strains no longer exhibited heteroresistance to CZA when the concentration of avibactam increases to 16 mg/L. These results suggest that increasing avibactam concentrations effectively prevents the emergence of CZA heteroresistance. Zhang et al. found that increasing avibactam concentrations to 8 mg/L could also restore the susceptibility to CZA in low-level CZA resistant strains (Zhang et al., 2020). However, the combination of ceftazidime and avibactam is suitable as a fixed-dose combination ratio of 4:1 due to their complementary pharmacokinetic profiles (e.g., 2 h half-life) (Sy et al., 2019). The dosage determination for ceftazidime–avibactam combination was based on achieving sufficient coverage for the pharmacokinetic/pharmacodynamic (PK/PD) targets, thereby ensuring a safe and effective therapeutic option for the clinical treatment of infections (Sy et al., 2019). Therefore, it is difficult to adjust the concentration of avibactam for individual patients in the clinical practice as the drug is a combination formulation with fixed ratios. The inhibitory effects of combination therapy on CZA heteroresistance were then investigated. We found that the CZA heteroresistance rate significantly decreased after combining CZA with 0.5 × MIC tigecycline, suggesting that combined treatment with tigecycline is an effective strategy for inhibiting CZA heteroresistance in CRKP isolates. Notably, previous studies have shown that CZA improves the antibacterial efficacy of polymyxin B against KPC-producing *K. pneumoniae* and hinders the emergence of polymyxin B heteroresistance (Ma et al., 2019). However, in this study, polymyxin B did not inhibit the occurrence of CZA heteroresistance, suggesting that the inhibitory effect of combination therapy on heteroresistance may not be bidirectional. As the frequency of CZA heteroresistance in CRKP strains increased along with the MIC, CZA-tigecycline combination treatment was recommended to prevent CZA heteroresistance in cases where the CZA MIC was close to the resistance breakpoint.

In conclusion, this study identified the occurrence of CZA heteroresistance in CRKP isolates in China, with strains mainly belonging to ST11 clonality and producing KPC enzymes. High expression of KPC was found to be responsible for CZA heteroresistance in these CRKP strains. Increasing the concentration of avibactam or combining CZA with tigecycline could effectively prevent the development of CZA heteroresistance in these CRKP isolates. Combination therapy might be the preferred choice for treating strains with CZA MICs nearing the resistance breakpoint. Besides, imipenem-relebactam and meropenem-vaborbactam may serve as alternative therapeutic options when clinical isolates with CZA heteroresistance are detected.

## Data Availability

The datasets presented in this study can be found in online repositories. The names of the repository/repositories and accession number(s) can be found in the article/**Supplementary Material**.
