# Supplementary material for: Overexpression of KPC contributes to ceftazidime-avibactam heteroresistance in clinical isolates of carbapenem-resistant Klebsiella pneumoniae
Source: Front Cell Infect Microbiol. 2024 Dec 6;14:1450530. doi: 10.3389/fcimb.2024.1450530 (PMC11659205; doi:10.3389/fcimb.2024.1450530)
Supplement: Supplementary file 1 [file DataSheet1.docx]

Supplementary Material

Overexpression of KPC contributes to ceftazidime-avibactam heteroresistance in clinical isolates of carbapenem-resistant *Klebsiella pneumoniae*

**Yitan Li^1+^, Xiandi Chen^1+^, Yingyi Guo^2+^, Yingzhuo Lin^1^, Xiaohu Wang^1^, Guohua He^1^, Mingzhen Wang^1^, Jianbo Xu^1^, Mingdong Song^1^, Xixi Tan^1^, Chao Zhuo^2*^, Zhiwei Lin^1*^**

*** Correspondence:** Zhi-wei Lin: 422156321@qq.com and Chao Zhuo: chao_sheep@263.net

# Supplementary Figures and Tables

## Supplementary Figures


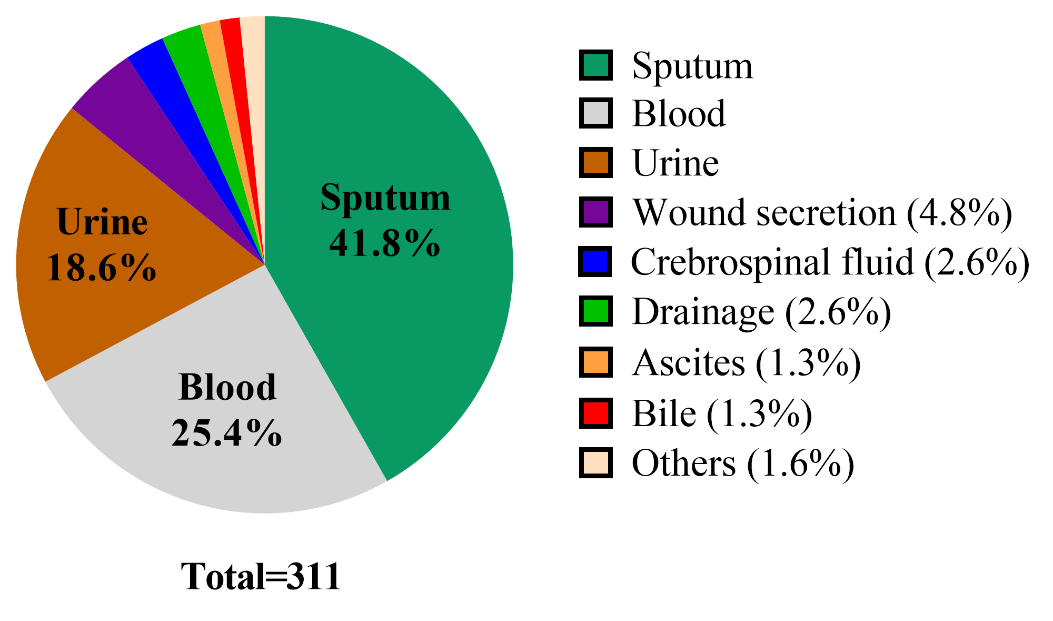


**Supplementary Figure 1.** The distribution of sample source among *K. pneumoniae*. Besides the sources described above, other source included abscess fluid (2 strains), bronchoalveolar lavage fluid (2 strains), throat swabs (1 strain).


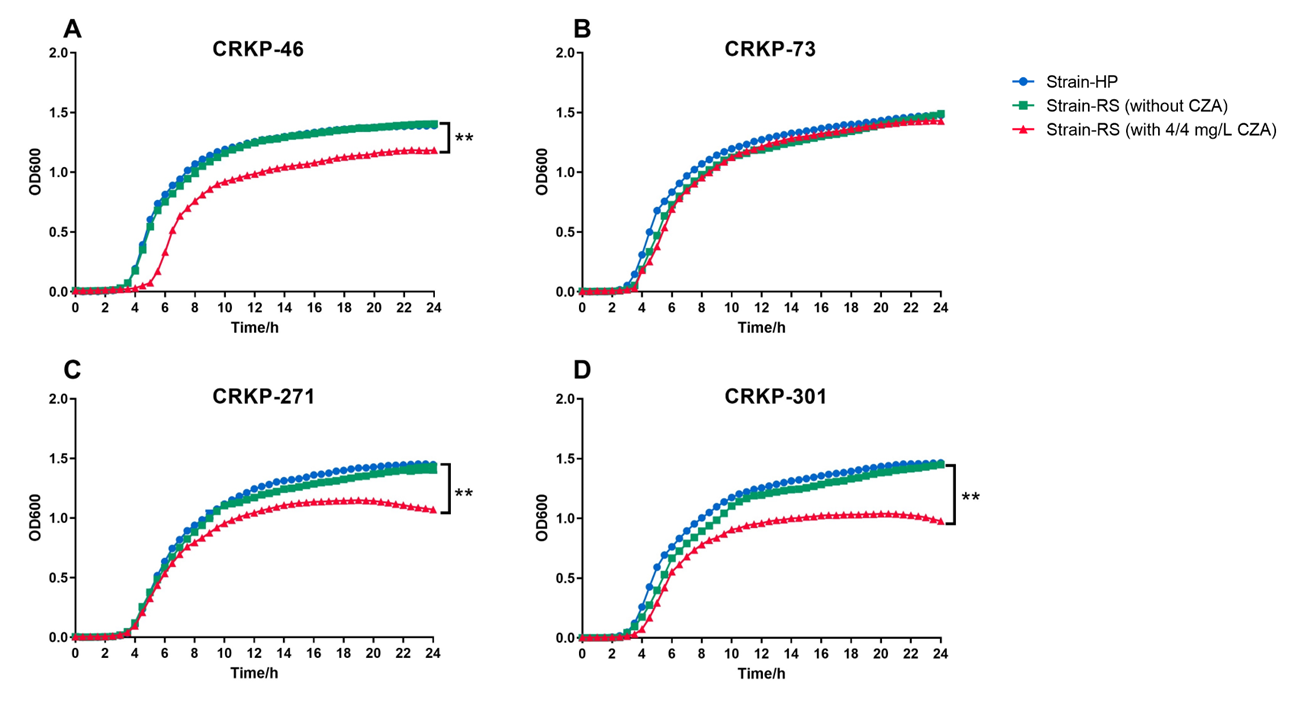


**Supplementary Figure 2.** Growth curves in four groups of randomly selected resistant subpopulations and their parental strains cultured with and without CZA pressure. Overnight bacterial cultures of resistant subpopulations and their parental strains were 1:200 diluted into MHB containing 4/4 mg/L CZA or MHB separately and incubated at 37℃ with shaking at 220 rpm. The OD600 was measured every 30 min. Values are means ± standard deviations from 3 independent wells. A repeated measures analysis of variance (ANOVA) was used to assess the statistical significance. **: *P*<0.05. Strain-HP: CZA heteroresistant parental strain. Strain-RS (without CZA): resistant subpopulations of CZA heteroresistant strains cultured without CZA pressure. Strain-RS (with 4/4 mg/L CZA): resistant subpopulations of CZA heteroresistant strains cultured with 4/4 mg/L CZA.


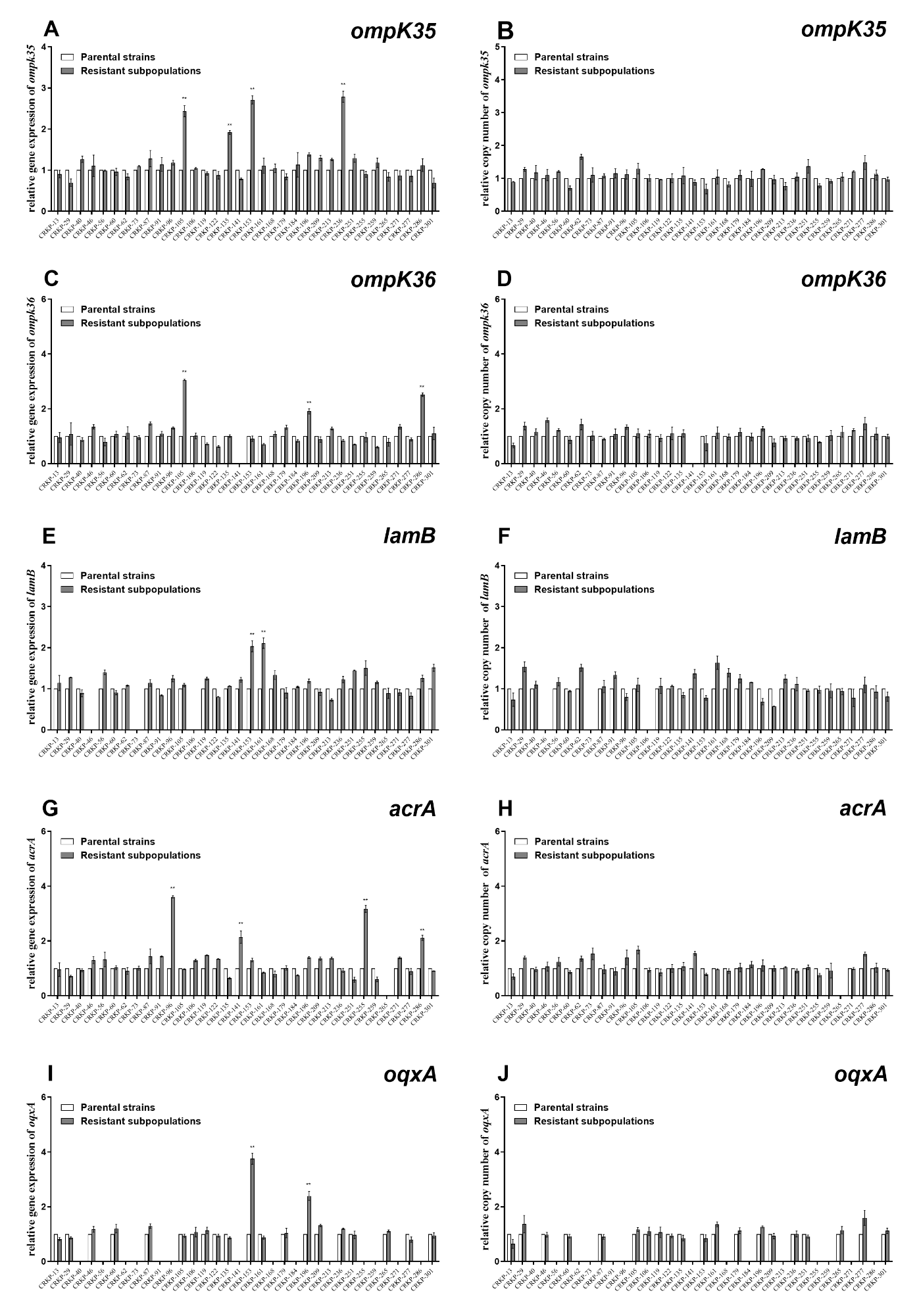


**Supplementary Figure 3.** Relative expression level and copy number of CZA resistance genes in resistant subpopulations compared with their parental strains. The parental strains and resistant subpopulations were grown in LB to the logarithmic phase for RNA extraction and to the stationary phase for DNA extraction. Total RNA or total DNA were extracted. The expression level and copy number of *ompK35* (A, B), *ompK36* (C, D), *lamB* (E, F), *acrA* (G, H), *and oqxA* (I, J) were then examined by qRT-PCR. The housekeeping gene, *rrsE,* was used as the endogenous reference gene. The parental strains were used as the reference strain (expression level = 1, copy number = 1). All experiments were carried out in triplicate. **: *P*<0.05.


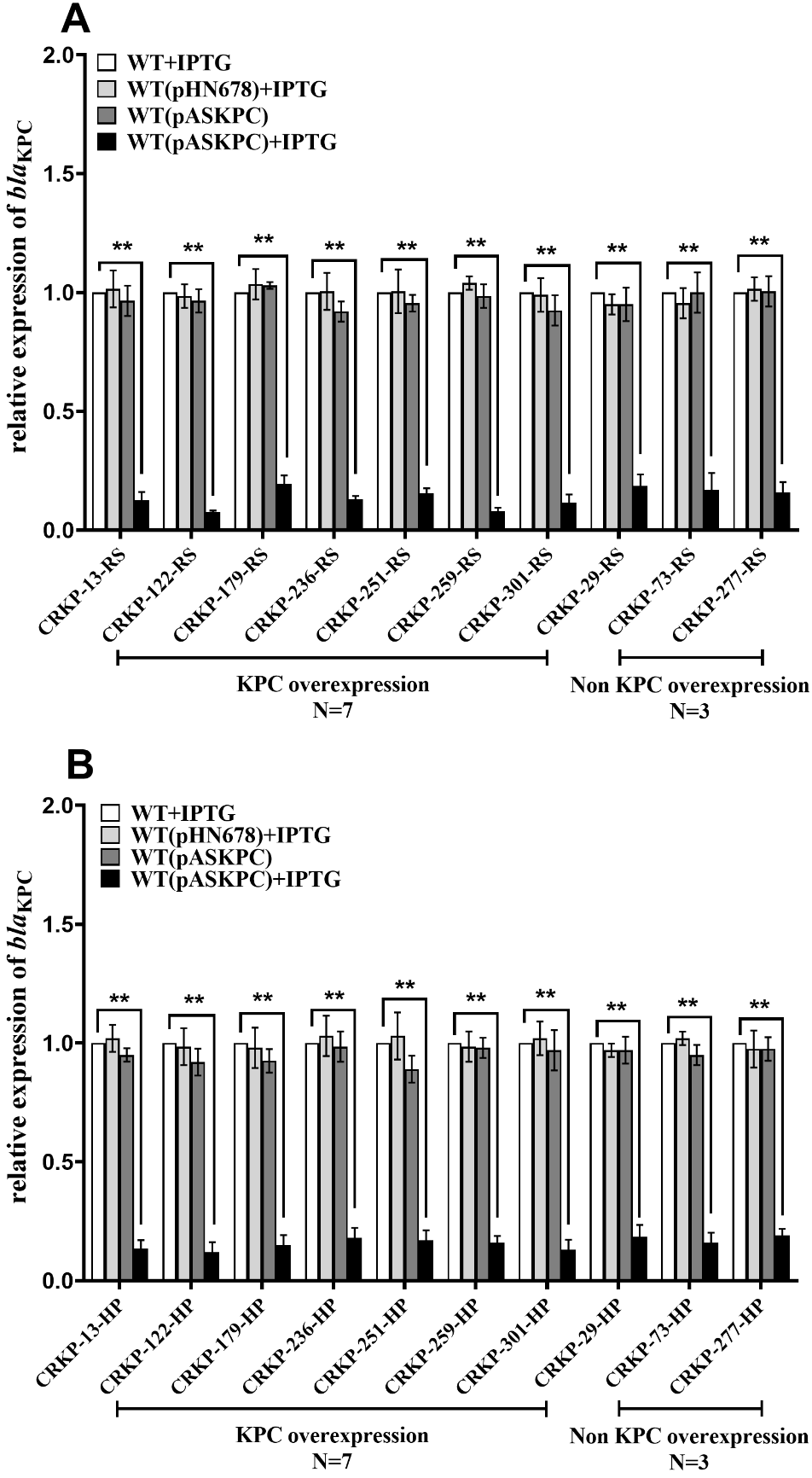


**Supplementary Figure 4.** Expression of *bla*_KPC_ in asRNA silencing strains. Resistant subpopulations and parental strains transformed with pASKPC were grown in LB to the logarithmic phase and induced with or without 1 mM IPTG. Total RNA was extracted and the expressions of *bla*_KPC_ in resistant subpopulations (**A**) or parental strains (**B**) were assessed by qRT-PCR. The housekeeping gene, *rrsE,* was used as an endogenous reference gene. Each wild-type strain was used as the reference strain (expression = 1.0). All qRT-PCR experiments were carried out in triplicate. ** *P*<0.05. HP: CZA heteroresistant parental strain, RS: resistant subpopulations of CZA heteroresistant strains. WT: wild-type of the strains. WT (pHN678): strains transformed with pHN678 plasmid. WT (pASKPC): strains transformed with pASKPC plasmid. IPTG: isopropyl β-D-thiogalactoside.

## Supplementary Tables

**Supplementary Table 1.** Primers used for PCR in this study.

| **Target gene** | **Primer** | **Sequence (5'-3')** | **Product length (bp)** | **Source** |
| --- | --- | --- | --- | --- |
| **MLST determination** | | | | |
| *gapA* | gapA-F | TGAAATATGACTCCACTCACGG | 662 | (Diancourt et al., 2005) |
|  | gapA-R | CTTCAGAAGCGGCTTTGATGGCTT |  |  |
| *infB* | infB-F | CTCGCTGCTGGACTATATTCG | 462 | (Diancourt et al., 2005) |
|  | infB-R | CTCGCTGCTGGACTATATTCG |  |  |
| *mdh* | mdh-F | CCCAACTCGCTTCAGGTTCAG | 756 | (Diancourt et al., 2005) |
|  | mdh-R | CCGTTTTTCCCCAGCAGCAG |  |  |
| *pgi* | pgi-F | GAGAAAAACCTGCCTGTACTGCTGGC | 566 | (Diancourt et al., 2005) |
|  | pgi-R | CGCGCCACGCTTTATAGCGGTTAAT |  |  |
| *phoE* | phoE-F | ACCTACCGCAACACCGACTTCTTCGG | 602 | (Diancourt et al., 2005) |
|  | phoE-R | TGATCAGAACTGGTAGGTGAT |  |  |
| *rpoB* | rpoB-F | GGCGAAATGGCWGAGAACCA | 1075 | (Diancourt et al., 2005) |
|  | rpoB-R | GAGTCTTCGAAGTTGTAACC |  |  |
| *tonB* | tonB-F | CTTTATACCTCGGTACATCAGGTT | 539 | (Diancourt et al., 2005) |
|  | tonB-R | ATTCGCCGGCTGRGCRGAGAG |  |  |
| **Carbapenemase type determination** | | | | |
| *bla*_KPC_ | KPC-F | TGTCACTGTATCGCCGTC | 1011 | (Rasheed et al., 2008) |
|  | KPC-R | GTCAGTGCTCTACAGAAAACC |  |  |
| *bla*_NDM-1_ | NDM-1-F | GGTTTGGCGATCTGGTTTTC | 621 | (Candan and Aksoz, 2015) |
|  | NDM-1-R | CGGAATGGCTCATCACGATC |  |  |
| *bla*_OXA-48_ | OXA-48-F | GCGTGGTTAAGGATGAACAC | 438 | (Candan and Aksoz, 2015) |
|  | OXA-48-R | CATCAAGTTCAACCCAACCG |  |  |
| *bla*_IMP_ | IMP-F | GGAATAGAGTGGCTTAAYTCTC | 232 | (Candan and Aksoz, 2015) |
|  | IMP-R | GGTTTAAYAAAACAACCACC |  |  |
| *bla*_VIM_ | VIM-F | GATGGTGTTTGGTCGCATA | 390 | (Candan and Aksoz, 2015) |
|  | VIM-R | CGAATGCGCAGCACCAG |  |  |
| **Capsule serotype determination** | | | | |
| *wzi* | wzi-F | GTGCCGCGAGCGCTTTCTATCTTGGTATTCC | 580 | (Brisse et al., 2013) |
|  | wzi-R | GAGAGCCACTGGTTCCAGAAYTTSACCGC |  |  |
| **Mutations determination in CZA resistance genes** | | | | |
| *bla*_KPC_ | KPC-F | ACCCTTGCCATCCCGTGTGC | 2288 | (Livermore et al., 2015) |
|  | KPC-R | CGCCATCGTCAGTGCTCTAC |  |  |
| *ompK*35 | ompK35-F | GGATGGAAAGATGCCTTCAG | 1392 | (Clancy et al., 2013) |
|  | ompK35-R | CATGACGAGGTTCCATTGTG |  |  |
| *ompK*36 | ompK36-F | GGGAAGAATCGCACGAAATA | 1774 | (Clancy et al., 2013) |
|  | ompK36-R | TCTTACCAGGGCGACAAGAG |  |  |
| *mdrA* (PBP2) | mdrA-F | TGATGATCAGGGTAGACTAAG | 1974 | This study |
|  | mdrA-R | GATGGATTTTATCCCATAGCG |  |  |
| *ftsI* (PBP3) | ftsI-F | GATGCAACACGTCGATCCATC | 1873 | This study |
|  | ftsI-R | TCCGGTGCGTTTGGCACCCAT |  |  |
| *l*amB | lamB-F | ATGTCCTTAGGTATTCGACC | 1736 | This study |
|  | lamB-R | AGCGGTAGCCAGGCAATAGC |  |  |

**Supplementary Table 2.** Primers for qRT-PCR in this study.

| **Target** | **Primer^a^** | **Sequence (5'-3')** | **Product Length (bp)** | **Source or reference** |
| --- | --- | --- | --- | --- |
| *rrsE* | qrrsE-F | CTACAATGGCATATACAA | 130 | This study |
|  | qrrsE-R | TTCTGATCTACGATTACT |  |  |
| *bla*_KPC_ | qKPC-F | GACACACCCATCCGTTAC | 79 | This study |
|  | qKPC-R | TCATGCCTGTTGTCAGATAT |  |  |
| *ompK35* | qompK35-F | AATATGACGCTAACAACA | 79 | This study |
|  | qompK35-R | AAGTGGTTATCTTCTTCC |  |  |
| *ompK36* | qompK36-F | AGACTTACAACGCAACTC | 89 | This study |
|  | qompK36-R | GTACTGAGCAACCACTTC |  |  |
| *lamB* | qlamB-F | GTCTTGGTAACGAATGTG | 122 | This study |
|  | qlamB-R | GTCATCTTCCTGATTCAC |  |  |
| *acrA* | qacrA-F | GGCAAACATGGATCAACTG | 97 | This study |
|  | qacrA-R | GGCGGTATCGTAGTCTTG |  |  |
| *oqxA* | qoqxA-F | CGCAGCTTAACCTCGACTTCA | 103 | This study |
|  | qoqxA-R | ACACCGTCTTCTGCGAGACC |  |  |

^a^Primers for qRT-PCR were designed by Beacon designer software 8.14.

**Supplementary Table 3.** Bacterial strains and plasmids used in the *bla*_KPC_ asRNA silencing experiment.

| **Name** | **Description** | **Source or reference** |
| --- | --- | --- |
| **Bacterial strains** |  |  |
| *K. pneumoniae* | | |
| CRKP-13-HP | CZA heteroresistant parental isolate CRKP-13 | People’s Hospital of Yangjiang |
| CRKP-122-HP | CZA heteroresistant parental isolate CRKP-122 | People’s Hospital of Yangjiang |
| CRKP-179-HP | CZA heteroresistant parental isolate CRKP-179 | People’s Hospital of Yangjiang |
| CRKP-236-HP | CZA heteroresistant parental isolate CRKP-236 | People’s Hospital of Yangjiang |
| CRKP-251-HP | CZA heteroresistant parental isolate CRKP-251 | People’s Hospital of Yangjiang |
| CRKP-259-HP | CZA heteroresistant parental isolate CRKP-259 | People’s Hospital of Yangjiang |
| CRKP-301-HP | CZA heteroresistant parental isolate CRKP-301 | People’s Hospital of Yangjiang |
| CRKP-29-HP | CZA heteroresistant parental isolate CRKP-29 | People’s Hospital of Yangjiang |
| CRKP-73-HP | CZA heteroresistant parental isolate CRKP-73 | People’s Hospital of Yangjiang |
| CRKP-277-HP | CZA heteroresistant parental isolate CRKP-277 | People’s Hospital of Yangjiang |
| CRKP-13-RS | Resistance suspopulation of CZA heteroresistant isolate CRKP-13 | this study |
| CRKP-122-RS | Resistance suspopulation of CZA heteroresistant isolate CRKP-122 | this study |
| CRKP-179-RS | Resistance suspopulation of CZA heteroresistant isolate CRKP-179 | this study |
| CRKP-236-RS | Resistance suspopulation of CZA heteroresistant isolate CRKP-236 | this study |
| CRKP-251-RS | Resistance suspopulation of CZA heteroresistant isolate CRKP-251 | this study |
| CRKP-259-RS | Resistance suspopulation of CZA heteroresistant isolate CRKP-259 | this study |
| CRKP-301-RS | Resistance suspopulation of CZA heteroresistant isolate CRKP-301 | this study |
| CRKP-29-RS | Resistance suspopulation of CZA heteroresistant isolate CRKP-29 | this study |
| CRKP-73-RS | Resistance suspopulation of CZA heteroresistant isolate CRKP-73 | this study |
| CRKP-277-RS | Resistance suspopulation of CZA heteroresistant isolate CRKP-277 | this study |
| CRKP-13-HP (pASKPC) | CRKP-13-HP introduced with plasmid pASKPC | this study |
| CRKP-122-HP (pASKPC) | CRKP-122-HP introduced with plasmid pASKPC | this study |
| CRKP-179-HP (pASKPC) | CRKP-179-HP introduced with plasmid pASKPC | this study |
| CRKP-236-HP (pASKPC) | CRKP-236-HP introduced with plasmid pASKPC | this study |
| CRKP-251-HP (pASKPC) | CRKP-251-HP introduced with plasmid pASKPC | this study |
| CRKP-259-HP (pASKPC) | CRKP-259-HP introduced with plasmid pASKPC | this study |
| CRKP-301-HP (pASKPC) | CRKP-301-HP introduced with plasmid pASKPC | this study |
| CRKP-29-HP (pASKPC) | CRKP-29-HP introduced with plasmid pASKPC | this study |
| CRKP-73-HP (pASKPC) | CRKP-73-HP introduced with plasmid pASKPC | this study |
| CRKP-277-HP (pASKPC) | CRKP-277-HP introduced with plasmid pASKPC | this study |
| CRKP-13-RS (pASKPC) | CRKP-13-RS introduced with plasmid pASKPC | this study |
| CRKP-122-RS (pASKPC) | CRKP-122-RS introduced with plasmid pASKPC | this study |
| CRKP-179-RS (pASKPC) | CRKP-179-RS introduced with plasmid pASKPC | this study |
| CRKP-236-RS (pASKPC) | CRKP-236-RS introduced with plasmid pASKPC | this study |
| CRKP-251-RS (pASKPC) | CRKP-251-RS introduced with plasmid pASKPC | this study |
| CRKP-259-RS (pASKPC) | CRKP-259-RS introduced with plasmid pASKPC | this study |
| CRKP-301-RS (pASKPC) | CRKP-301-RS introduced with plasmid pASKPC | this study |
| CRKP-29-RS (pASKPC) | CRKP-29-RS introduced with plasmid pASKPC | this study |
| CRKP-73-RS (pASKPC) | CRKP-73-RS introduced with plasmid pASKPC | this study |
| CRKP-277-RS (pASKPC) | CRKP-277-RS introduced with plasmid pASKPC | this study |
| *E. coli* |  |  |
| DH5α | *supE44 △lacU169 hsdR17 recA1 endA1 gyrA96 thi-1 relA1* | Invitrogen |
| **Plasmids** |  |  |
| pHN678 | *P_trc_* - MCS-PT7 - *T_rrnB_*, *lacI^q^*, p15A *ori*, IPTG induced, *Cm*^Ra^ | (Nakashima et al., 2006) |
| pASKPC | pHN678 inserted with the asRNA silencing sequence of *bla*_KPC_ | this study |

^a^*Cm*^R^: chloramphenicol resistance.

**Supplementary Table 4.** Primers used for *bla*_KPC_ asRNA silencing experiment.

| **Primers** | **Sequences（5’-3’）** | **Product**  **Length (bp)** | **Underline** |
| --- | --- | --- | --- |
| **Construction of the *bla_KPC_* silencing plasmids** | | | |
| ASKPC-F | CCCAAGCTTTTCAAACAAGGAATATCGTTG | 158 | HindIII |
| ASKPC-R | CGCGGATCCAAGTCCTGTTCGAGTTTAGCG |  | BamHI |
| **Verification of the *bla_KPC_* silencing plasmids** | | | |
| IDASKPC-F | GTATCGCCGTCTAGTTCTGCT | 460 |  |
| IDASKPC-R | CGTTACTGGTTTCACATTCAC |  |  |

**Supplementary Table 5.** Changes of CZA MICs in resistant subpopulations of heteroresistance isolates after 30 passages in antibiotic-free medium.

| **Strain** | **CZA MIC (mg/L)** | | | | | | |
| --- | --- | --- | --- | --- | --- | --- | --- |
|  | **Parental** | **5 passages** | **10 passages** | **15 passages** | **20 passages** | **25 passages** | **30 passages** |
| CRKP-46-RS^a^ | 16/4 | 8/4 | 4/4 | 4/4 | 4/4 | 4/4 | 4/4 |
| CRKP-60-RS | 32/4 | 16/4 | 8/4 | 8/4 | 4/4 | 4/4 | 4/4 |
| CRKP-73-RS | 32/4 | 16/4 | 8/4 | 8/4 | 8/4 | 8/4 | 8/4 |
| CRKP-91-RS | 16/4 | 8/4 | 8/4 | 4/4 | 4/4 | 4/4 | 4/4 |
| CRKP-135-RS | 16/4 | 8/4 | 8/4 | 4/4 | 4/4 | 4/4 | 2/4 |
| CRKP-161-RS | 32/4 | 8/4 | 8/4 | 8/4 | 8/4 | 8/4 | 4/4 |
| CRKP-236-RS | 16/4 | 8/4 | 4/4 | 4/4 | 4/4 | 4/4 | 4/4 |
| CRKP-259-RS | 16/4 | 16/4 | 8/4 | 8/4 | 4/4 | 4/4 | 4/4 |
| CRKP-271-RS | 16/4 | 8/4 | 4/4 | 4/4 | 4/4 | 4/4 | 4/4 |
| CRKP-301-RS | 32/4 | 16/4 | 8/4 | 8/4 | 8/4 | 8/4 | 8/4 |

^a^RS, resistant subpopulation of CZA heteroresistant isolates.

**Supplementary Table 6.** Mutations identified in heteroresistant subpopulations compared to their parental strains by WGS.

| **Strains** | **Proteins (Mutations)** | **Description** | **Predicted Function** | **Source** |
| --- | --- | --- | --- | --- |
| CRKP-56-RS | RsxC (VK688AE) | Ion-translocating oxidoreductase complex subunit C | The resistance to oxidative | (Moreau and Loiseau, 2016) |
|  |  |  |  |  |
| CRKP-62-RS | RffH (I5V) | Glucose-1-phosphate thymidylyltransferase 2 | Regulate the dTDP-glucose production | (Rao and Kuzminov, 2020) |
|  |  |  |  |  |
| CRKP-87-RS | RffH (I5V) | Glucose-1-phosphate thymidylyltransferase 2 | Regulate the dTDP-glucose production | (Rao and Kuzminov, 2020) |
|  | RmlB (D48E) | dTDP-glucose 4,6-dehydratase | dTDP-rhamnose synthesis enzyme | (van der Beek et al., 2019) |
|  | DgcP (I248L) | Diguanylate cyclase DgcP | involved in bacterial biofilm formation and motilities | (Nicastro et al., 2020) |
|  |  |  |  |  |
| CRKP-179-RS | RffH (I5V) | Glucose-1-phosphate thymidylyltransferase 2 | Regulate the dTDP-glucose production | (Rao and Kuzminov, 2020) |
|  | RmlB (D48E) | dTDP-glucose 4,6-dehydratase | dTDP-rhamnose synthesis enzyme | (van der Beek et al., 2019) |
|  |  |  |  |  |
| CRKP-277-RS | RffH (I5V) | Glucose-1-phosphate thymidylyltransferase 2 | Regulate the dTDP-glucose production | (Rao and Kuzminov, 2020) |
|  | RmlB (D48E) | dTDP-glucose 4,6-dehydratase | dTDP-rhamnose synthesis enzyme | (van der Beek et al., 2019) |

^a^Mutations in heteroresistant subpopulations were identified by comparing them with their parental strains.

**Supplementary Table 7.** The frequency of CZA heteroresistance in *bla*_KPC_-silenced parental strains.

| **Transformed**  **plasmid** | **Isolates** |  | **PAP test results^a^** | | |
| --- | --- | --- | --- | --- | --- |
|  |  |  | **Wild-type strain^b^** | **Vector control strain** | **Derivative strain** |
| **KPC overexpression strains** | | | | | |
| pASKPC | CRKP-13 |  | + | + | – |
|  | CRKP-122 |  | + | + | – |
|  | CRKP-179 |  | + | + | – |
|  | CRKP-236 |  | + | + | – |
|  | CRKP-251 |  | + | + | – |
|  | CRKP-259 |  | + | + | – |
|  | CRKP-301 |  | + | + | – |
| **Non KPC overexpression strains** | | | | | |
| pASKPC | CRKP-29 |  | + | + | + |
|  | CRKP-73 |  | + | + | + |
|  | CRKP-277 |  | + | + | + |

^a^CZA heteroresistance is defined as an CZA-susceptible isolate (CZA MIC of ≤ 8/4 mg/L) with subpopulations growing in the presence ≥ 16/4 mg/L CZA induced with 1 mM IPTG, with a detection threshold of 20 CFU/mL. +: positive; –: negative.

^b^Wild-type strain: CZA heteroresistant parental strains. Vector control strain: wild-type strain transformed with pHN678. Derivative strain: wild-type strain transformed with pASKPC.

**Supplementary Table 8.** Effects of different avibactam concentrations on CZA MICs.

| **Strains** | **CZA MIC (mg/L）** | | | | |
| --- | --- | --- | --- | --- | --- |
|  | **AVI^a^: 4** **mg/L** | **AVI: 8** **mg/L** | **Fold change^b^** | **AVI: 16** **mg/L** | **Fold change** |
| CRKP-13-RS | 16 | 4 | 4^c^ | 0.125 | 128 |
| CRKP-29-RS | 16 | 8 | 2 | 0.125 | 128 |
| CRKP-40-RS | 16 | 0.25 | 64 | 0.25 | 64 |
| CRKP-46-RS | 16 | 4 | 4 | 0.125 | 128 |
| CRKP-56-RS | 16 | 0.5 | 32 | 0.125 | 128 |
| CRKP-60-RS | 32 | 32 | 1 | 0.5 | 64 |
| CRKP-62-RS | 16 | 4 | 4 | 1 | 16 |
| CRKP-73-RS | 32 | 16 | 2 | 16 | 2 |
| CRKP-87-RS | 32 | 1 | 32 | 0.125 | 256 |
| CRKP-91-RS | 16 | 4 | 4 | 0.125 | 128 |
| CRKP-96-RS | 16 | 4 | 4 | 0.125 | 128 |
| CRKP-105-RS | 32 | 8 | 4 | 2 | 16 |
| CRKP-106-RS | 16 | 8 | 2 | 0.125 | 128 |
| CRKP-119-RS | 32 | 8 | 4 | 4 | 8 |
| CRKP-122-RS | 32 | 2 | 16 | 2 | 16 |
| CRKP-135-RS | 16 | 4 | 4 | 2 | 8 |
| CRKP-141-RS | 64 | 16 | 4 | 0.5 | 128 |
| CRKP-153-RS | 32 | 0.25 | 128 | 0.25 | 128 |
| CRKP-161-RS | 32 | 32 | 1 | 0.25 | 128 |
| CRKP-168-RS | 16 | 4 | 4 | 1 | 16 |
| CRKP-179-RS | 32 | 16 | 2 | 0.25 | 128 |
| CRKP-184-RS | 16 | 4 | 4 | 0.5 | 32 |
| CRKP-196-RS | 32 | 8 | 4 | 0.25 | 128 |
| CRKP-209-RS | 64 | 16 | 4 | 1 | 64 |
| CRKP-213-RS | 32 | 8 | 4 | 8 | 4 |
| CRKP-236-RS | 16 | 8 | 2 | 2 | 8 |
| CRKP-251-RS | 16 | 8 | 2 | 0.125 | 128 |
| CRKP-255-RS | 32 | 4 | 8 | 0.5 | 64 |
| CRKP-259-RS | 16 | 0.125 | 128 | 0.125 | 128 |
| CRKP-265-RS | 32 | 8 | 4 | 1 | 32 |
| CRKP-271-RS | 16 | 4 | 4 | 0.125 | 128 |
| CRKP-277-RS | 32 | 8 | 4 | 2 | 16 |
| CRKP-286-RS | 64 | 16 | 4 | 0.5 | 128 |
| CRKP-301-RS | 32 | 8 | 4 | 0.5 | 64 |

^a^AVI: avibactam.

^b^The fold change means the ratio of CZA MIC with avibactam concentration is 8 mg/L (or 16 mg/L) and CZA MIC with avibactam concentration is 4 mg/L.

^c^Significant inhibition was defined as a ≥4-fold decrease in the MICs.

**Supplementary Table 9.** The Copy number of *bla*_KPC_ in resistant subpopulations with high expression of *bla*_KPC._

| **Isolates** | **Expression ratio of *bla*_KPC_^a^** | **Copy number ratio of *bla*_KPC_** |
| --- | --- | --- |
| CRKP-13-RS | 5.66±0.42 | 2.57±0.16 ⇧^b^ |
| CRKP-40-RS | 2.67±0.16 | 1.19±0.19 |
| CRKP-87-RS | 3.12±0.20 | 1.92±0.14 ⇧ |
| CRKP-96-RS | 2.98±0.10 | 2.34±0.30 ⇧ |
| CRKP-122-RS | 3.89±0.13 | 1.77±0.04 ⇧ |
| CRKP-179-RS | 3.84±0.16 | 1.98±0.23 ⇧ |
| CRKP-196-RS | 2.28±0.10 | 2.16±0.28 ⇧ |
| CRKP-209-RS | 2.55±0.18 | 1.05±0.16 |
| CRKP-213-RS | 3.62±0.30 | 2.09±0.22 ⇧ |
| CRKP-236-RS | 9.92±0.06 | 3.48±0.29 ⇧ |
| CRKP-251-RS | 4.15±0.39 | 0.87±0.03 |
| CRKP-259-RS | 4.94±0.33 | 2.75±0.24 ⇧ |
| CRKP-265-RS | 2.21±0.07 | 1.20±0.09 |
| CRKP-271-RS | 4.18±0.18 | 2.09±0.18 ⇧ |
| CRKP-286-RS | 2.71±0.07 | 1.75±0.04 ⇧ |
| CRKP-301-RS | 4.76±0.09 | 2.09±0.18 ⇧ |

^a^The expression level or copy number ratio of *bla*_KPC_ in resistant subpopulations was compared to the parental strains.

^b^⇧: The copy number of *bla*_KPC_ was significantly upregulated in resistant subpopulations compared with the parental strains.

# References

Brisse, S., Passet, V., Haugaard, A.B., Babosan, A., Kassis-Chikhani, N., Struve, C., et al. (2013). wzi Gene sequencing, a rapid method for determination of capsular type for *Klebsiella* strains. J Clin Microbiol 51(12)**,** 4073-4078. https://doi.org/10.1128/JCM.01924-13.

Candan, E.D., and Aksoz, N. (2015). *Klebsiella pneumoniae*: characteristics of carbapenem resistance and virulence factors. Acta Biochim Pol 62(4)**,** 867-874. https://doi.org/10.18388/abp.2015_1148.

Clancy, C.J., Chen, L., Hong, J.H., Cheng, S., Hao, B., Shields, R.K., et al. (2013). Mutations of the ompK36 porin gene and promoter impact responses of sequence type 258, KPC-2-producing *Klebsiella pneumoniae* strains to doripenem and doripenem-colistin. Antimicrob Agents Chemother 57(11)**,** 5258-5265. https://doi.org/10.1128/AAC.01069-13.

Diancourt, L., Passet, V., Verhoef, J., Grimont, P.A., and Brisse, S. (2005). Multilocus sequence typing of *Klebsiella pneumoniae* nosocomial isolates. J Clin Microbiol 43(8)**,** 4178-4182. https://doi.org/10.1128/JCM.43.8.4178-4182.2005.

Livermore, D.M., Warner, M., Jamrozy, D., Mushtaq, S., Nichols, W.W., Mustafa, N., et al. (2015). In vitro selection of ceftazidime-avibactam resistance in *Enterobacteriaceae* with KPC-3 carbapenemase. Antimicrob Agents Chemother 59(9)**,** 5324-5330. https://doi.org/10.1128/AAC.00678-15.

Nakashima, N., Tamura, T., and Good, L. (2006). Paired termini stabilize antisense RNAs and enhance conditional gene silencing in *Escherichia coli*. Nucleic Acids Res 34(20)**,** e138. https://doi.org/10.1093/nar/gkl697.

Rasheed, J.K., Biddle, J.W., Anderson, K.F., Washer, L., Chenoweth, C., Perrin, J., et al. (2008). Detection of the Klebsiella pneumoniae carbapenemase type 2 Carbapenem-hydrolyzing enzyme in clinical isolates of Citrobacter freundii and K. oxytoca carrying a common plasmid. J Clin Microbiol 46(6), 2066-2069. https://doi.org/10.1128/JCM.02038-07.Moreau, P.L., and Loiseau, L. (2016). Characterization of acetic acid-detoxifying Escherichia coli evolved under phosphate starvation conditions. Microb Cell Fact 15, 42. doi: 10.1186/s12934-016-0441-7.

Nicastro, G.G., Kaihami, G.H., Pulschen, A.A., Hernandez-Montelongo, J., Boechat, A.L., de Oliveira Pereira, T., et al. (2020). c-di-GMP-related phenotypes are modulated by the interaction between a diguanylate cyclase and a polar hub protein. Sci Rep 10(1), 3077. doi: 10.1038/s41598-020-59536-9.

Rao, T.V.P., and Kuzminov, A. (2020). Exopolysaccharide defects cause hyper-thymineless death in Escherichia coli via massive loss of chromosomal DNA and cell lysis. Proc Natl Acad Sci U S A 117(52), 33549-33560. doi: 10.1073/pnas.2012254117.

van der Beek, S.L., Zorzoli, A., Çanak, E., Chapman, R.N., Lucas, K., Meyer, B.H., et al. (2019). Streptococcal dTDP-L-rhamnose biosynthesis enzymes: functional characterization and lead compound identification. Mol Microbiol 111(4), 951-964. doi: 10.1111/mmi.14197.
